# Unlocking information for coordination of care in Australia: a qualitative study of information continuity in four primary health care models

**DOI:** 10.1186/1471-2296-14-34

**Published:** 2013-03-13

**Authors:** Michelle Banfield, Karen Gardner, Ian McRae, James Gillespie, Robert Wells, Laurann Yen

**Affiliations:** 1 Australian Primary Health Care Research Institute, Level 1, Ian Potter House, Cnr Gordon & Marcus Clarke Sts, The Australian National University, Canberra, ACT 0200, Australia; 2 Menzies Centre for Health Policy, School of Public Health, University of Sydney, Sydney, NSW 2006, Australia

**Keywords:** Care coordination, Information continuity, Chronic disease management, Primary health care

## Abstract

**Background:**

Coordination of care is considered a key component of patient-centered health care systems, but is rarely defined or operationalised in health care policy. Continuity, an aspect of coordination, is the patient’s experience of care over time, and is often described in terms of three dimensions: information, relational and management continuity. With the current health policy focus on both the use of information technology and care coordination, this study aimed to 1) explore how information continuity supports coordination and 2) investigate conditions required to support information continuity.

**Methods:**

Four diverse Australian primary health care initiatives were purposively selected for inclusion in the study. Each has improved coordination as an aim or fundamental principle. Each organization was asked to identify practitioners, managers and decision makers who could provide insight into the use of information for care coordination to participate in the study. Using in-depth semi-structured interviews, we explored four questions covering the scope and use of information, the influence of governance, data ownership and confidentiality and the influence of financial incentives and quality improvement on information continuity and coordination. Data were thematically analyzed using NVivo 8.

**Results:**

The overall picture that emerged across all four cases was that whilst accessibility and continuity of information underpin effective care, they are not sufficient for coordination of care for complex conditions. Shared information reduced unnecessary repetition and provided health professionals with the opportunity to access records of care from other providers, but participants described their role in coordination in terms of the active involvement of a person in care rather than the passive availability of information. Complex issues regarding data ownership and confidentiality often hampered information sharing. Successful coordination in each case was associated with responsiveness to local rather than system level factors.

**Conclusions:**

The availability of information is not sufficient to ensure continuity for the patient or coordination from the systems perspective. Policy directed at information continuity must give consideration to the broader ‘fit’ with management and relational continuity and provide a broad base that allows for local responsiveness in order for coordination of care to be achieved.

## Background

Coordination of care, incorporating factors such as inter-sectoral collaboration and facilitation of access, is one of the key components of health systems focused on patient-centered care [1]. There is good evidence that coordination is beneficial both for processes of patient care and patient outcomes [2]. However, previous research also suggests that coordination is inconsistently defined: for example, McDonald and colleagues identified more than 40 definitions of care coordination, the scope and characteristics of which varied according to their audience and purpose. Their working definition stated that care coordination is:

“…*the deliberate organization of patient care activities between two or more participants (including the patient) involved in a patient’s care to facilitate the appropriate delivery of health care services. Organizing care involves the marshalling of personnel and other resources needed to carry out all required patient care activities, and is often managed by the exchange of information among participants responsible for different aspects of care.” p.41*[2]

However, the authors recognized that their broad approach chosen for synthesizing the definitions may not suit all purposes and that narrower or closely related concepts may better suit particular circumstances [2]. One such area is the formulation of health policy: the mechanisms by which coordination is to be achieved are poorly understood and rarely identified in relevant policies [3-7]. For example, coordination of care is described in Australian health policy as a key principle for chronic disease management, with associated action areas and key directions [8], but guidance on what is meant by care coordination in this context and how it is expected to be achieved is absent.

Continuity of care, on the other hand, has been identified as one important aspect of coordination and one whose elements can be identified and measured [9,10]. Like coordination more broadly, continuity of care is associated with better outcomes such as higher satisfaction and lower hospitalization rates [11-13]. Waibel and colleagues suggest that the relationship between coordination and continuity is one of perspective: coordination reflects the provider and health system perspective, whereas continuity is what is experienced by the patient over time [13]. Reid and colleagues express a similar view, suggesting that a focus on relationships *between* providers reflects coordination, but in addition to patients they allow for providers to experience continuity by having the information needed to provide care and work with other providers [10].

Research examining continuity has identified three dimensions: information continuity, management continuity and relational continuity [9,10]. In primary care, continuity has often been seen in terms of relational continuity [e.g., [1,12], but as Bodenheimer notes, the increasing complexity of the care doctors are expected to provide means that it needs to extend beyond the relationship between a single provider and patient [14]. On this basis, information continuity will be the principal tool underpinning coordination since it is conducive to automation and systematization, and information itself can be made readily available [11].

In the Australian health care system, historical divisions of responsibility for funding and delivery between three levels of government have produced a complex mix of organizational and payment arrangements and this has given rise to service fragmentation and cost shifting [15]. In primary health care, there are few incentives for achieving continuity or coordination, especially across the spectrum of care required for prevention and between medical and allied health providers [15]. Funding for medical care is provided principally by fee-for-service and targeted incentive payments, whereas non-medical care such as that provided by allied health professionals is funded through state-based organizations or fee-for-service private practice [16]. In this context, there have been a number of government e-health and chronic disease initiatives aiming to improve continuity and coordination through information [8,17]. These include the Personally Controlled Electronic Health Record (PCEHR), a centralized electronic health record system designed to make health information easily but securely available to providers authorized by patients [17]. Similarly, the National Chronic Disease Strategy recognizes that the availability of good information for individual patients and at the population level is essential for achieving its objectives of enhancing prevention, maximizing the wellbeing of people with chronic illness and ensuring the health system can manage demand [8]. These initiatives have intended benefits both for better continuity and coordination of care for individuals and better policy development through the data that would potentially become available for use in research and planning [8,17]. What is not clear is what the current conditions are in the Australian context to enable continuity of information for the individual and coordination within the system. There is little understanding of how different organizations operating at different levels of the health system and with different goals and governance use information to support coordination. Without such an understanding, it is difficult to see how best to take advantage of the technological opportunities offered by increasing ease of electronic communication and data sharing to achieve better patient, or system, outcomes.

The purpose of this research was to explore information continuity in Australian primary care to assist decision makers in developing effective policy for coordination of care.

To do this, our aims were to:

1. Explore how information continuity operates to support coordination; and

2. Investigate what conditions are required to support information continuity in four different primary care organizations.

## Methods

Ethics approval for the study was provided by The Australian National University Human Research Ethics Committee.

### Study design

This study used in-depth semi-structured interviews to explore four key questions:

1. What is the scope of information used?

2. How is information used to support care?

3. How do factors such as governance arrangements influence the use of information?

4. What is the influence of financial incentives and quality improvement programs?

These questions were designed to explore continuity within each of four primary care organizations, using the Innovative Care for Chronic Conditions framework [18] to investigate micro (questions 1 and 2), meso (questions 2 and 3) and macro (question 4) level influences. This paper presents primary results for each organization, separated as cases to identify key findings for that organization type. Detailed analysis of information continuity against the framework is the subject of a separate forthcoming paper (Gardner et al. unpublished).

### Participants

Four diverse initiatives were purposively selected for inclusion in the study, as examples of different primary care business models operating within the Australian system (see Table 1). Organizations were included if they had improved coordination as an aim or fundamental principle and represented a common primary care model (e.g., private enterprise, regional organization). Each organization was asked to identify practitioners and managers who could provide insight into the use of information for care coordination to participate in the study.

**Table 1:** Case study organizations and participants

**Case**	**Type of organization**	**Professionals interviewed**
One	Network of fee-for-service practices and state-funded community health services. Operates a “hub and spoke” model for virtual integration of health care and community service providers. Targets people with complex needs.	GPs, regional managers, regional planners, nurses, community health managers
Two	Regional electronic information initiative for chronic illness management. Includes patient portal to access health information and test results.	Practice manager, administrator, nurse, diabetes educator, exercise physiologist.
Three	Large scale shared electronic health record (SEHR). Aims to provide better continuity of care for highly mobile Indigenous population. Consists of summary of medical records accessible to all health professionals at point of care.	Public health doctor, system manager
Four	National company engaged by government for provision of health promotion and illness prevention, triage, advice and referral for callers to a “health line”, chronic and mental illness management and workplace health.	GPs, nurses, program managers, clinical quality managers.

### Procedure

Interviews were conducted by three members of the research team by telephone or face-to-face during August, September and October 2011. Interviews were digitally recorded and supplementary notes were taken during the interviews. Written consent for participation and the recording were secured from all participants and confirmed verbally at the commencement of each interview. Participants were interviewed individually with the exception of four members of Case One who participated in a group interview and teleconference due to scheduling constraints. The duration of individual interviews ranged from 20 to 60 minutes and the group interview ran for 80 minutes. All interviews followed a semi-structured protocol consisting of four key questions and supplementary prompts (see Additional file 1).

### Data analysis

Interviews were transcribed verbatim and reviewed for accuracy. Computer-assisted thematic analysis using NVivo 8 software (QSR International) was conducted on the interview transcripts and notes. Two members of the research team independently coded the data using the interview questions and prompts as the initial framework. Inductive coding was also used where topics discussed were outside the initial framework. The final coding structure and themes were decided by discussion and consensus in team meetings. As a key interest was to investigate information continuity within each model, data were collated by case to identify key themes. Findings are presented by organization with supporting quotes where appropriate. Due to the small number of participants, sources are identified only by a unique number within the interviews conducted for that organization.

## Results

A total of 17 participants were interviewed across the four organizations, including nurses, allied health professionals, doctors and managers in practice, planning and quality improvement roles.

### Case One

As an initiative purposely designed to “virtually integrate” providers from different sections of the health system, primarily fee-for-service doctors and state-funded community health providers, the availability and transfer of information was a key part of Case One. As detailed below, participants felt that they were succeeding with their aim of improving care for patients with very complex needs. This was achieved through detailed collection and sharing of information, but success was primarily due to the management practices of the program’s nurses, who actively transferred information between providers.

### Scope of information used

Information routinely collected to assist with continuity of care included both health and social care information. A comprehensive assessment was completed when patients were referred to the initiative, which included clinical information such as diagnoses, demographics, risk behaviors such as smoking and social circumstances such as living situation. The assessment also included a plan for what the patient wanted to work on, which participants noted could function as a care plan.

The information collected in the assessment helped the program’s nurses to determine eligibility for community health services and for enrolment in the initiative. Should a patient not meet eligibility criteria, information would then be provided to the referring GP on alternative services that may be appropriate for the patient.

### How information is used to support care

One of the stated key objectives of Case One was to improve chronic disease management. The core components of the model of care included the integration of general practice and community health services. Patients and carers were considered to be at the centre of the model. Participants emphasized that the information shared was driven by the patient’s consent when they enroll in the initiative. Information sharing for the purposes of care planning and support for service access was a listed part the initiative’s activities. It became clear from participants that the effective flow of information relied on the liaison nurses to act as coordinators and facilitators. This was in large part through their ability to gain the trust of all parties and so act as a bridge for information to travel between the different services, and their effectiveness in fostering problem solving on behalf of patients with complex care needs. The nurses’ activities included conducting the eligibility assessments, providing information to GPs about enrolment in the initiative or other options for patients not eligible, organizing case conferences between patients, GPs, other health providers and community services involved in the patient’s care and helping to streamline the services being provided to patients. However, problems such as restricted access to records in hospital and community health computer systems, incompatible computer software, a lack of secure messaging or even a lack of computerization for some GPs meant that the nurses and/or the patients very often had to physically transport information between providers.

…we send the letter back to the GP saying we’ve got this happening, they’ve enrolled with [the initiative], would you like any information, would you like to do a case conference? And then we contact… I contact a week later to say, “Look, I sent you that info, what are you… are you happy with everything like that? Would you like to do anything? How is it from your side?” [C1I2]

### Influence of governance, confidentiality, data ownership and standards

The Case One initiative was overseen by a steering committee, consisting of representatives from the GPs, community health services and bureaucracy. This complex mix of traditionally disparate groups coupled with the difficulties in information flow identified above meant that issues such as data ownership were unclear. The information in individual systems was seen to belong to that organization, with common data managed by the liaison nurses day to day. The custodian of the combined data informing the initiative’s services was identified as the steering committee. However, the exchange of information between the organizations was inhibited by concerns about confidentiality by the individual organizations, particularly with respect to the use of these data to plan service developments. Despite endorsing population planning to target particular areas of disadvantage and particular groups, participants in all roles, clinical and managerial, felt there were still significant barriers to the sharing of data. Decisions about future service developments were generally based on aggregated data held at state or national level, and information from the initiative itself was not incorporated.

Participants particularly saw the lack of computerization and a secure messaging system to ensure confidentiality as inhibiting effective service provision. Not only was limited clinical information committed to electronic records in many practices, the lack of a secure method of transfer limited what could be shared and forced a reliance on face-to-face, telephone and fax transfer of records.

### Influence of financial incentives and quality improvement programs

Participants described keeping doctors abreast of Medicare Benefits Schedule (MBS) items that could be claimed for services such as case conferencing as part of the core practice support. Nurses and managers expected that financial incentives would be a useful way of motivating GPs to participate in extra activities, but it was found that money was not a great driver for participation. Instead, GPs were reported to be more interested in the benefit for the patients in participating in the initiative.

… we thought they’d be all interested in doing it to get the money, but they’re not that… they’re making enough money, and they’re quite happy… they said, “Well what’s the benefit … if there’s not going to be an increase of benefit to patients’ care, I’m not going to do that.” [C1I2]

There was some use of aggregated data for quality improvement processes and efficiency of the initiative overall. Effective use of the quality improvement data for planning was hampered by patchy uptake of the quality improvement programs and tools themselves. However, participants reported that internal evaluation data suggested that the initiative had resulted in substantial reductions for the number of bed days for some patients.

Overall, this suggests that financial incentives and quality improvement processes themselves were not having a large influence on the use of information for coordination in Case One. However, it appears that the increased information sharing and activities such as case conferencing and active case management that were core to the initiative did result in measurable effects on efficiency and quality.

### Other themes

In addition to the four core questions described in the study design above, some additional areas were explored to expand on participants’ responses to the main questions. One such area was how participants viewed the role of the initiative in coordination of care. Participants in Case One felt that their primary role to was facilitate communication between two areas of health, general practice and community health, in order to better serve a disadvantaged and underserviced community.

However, as already noted, this communication process and information sharing was heavily reliant on the members of the initiative working with one another to facilitate the sharing of data from state-funded and Commonwealth-funded sources and to facilitate care coordination.

### Case Two

The regional electronic record investigated for Case Two was supported by a meso level primary health care organization to improve access to and transfer of information between patients and their providers. The record was a detailed and useful tool for managing a considerable amount of patient information, particularly for chronic disease management, but similar to Case One, much of the transfer of information such as referrals still required manual intervention from practice staff.

### Scope of information used

The electronic record included a wide range of information to support clinical care and coordination, organized into a series of tabs to make the information easily accessible. Tabs included basic demographics, diagnoses, test results, medication lists and care plans, along with self-management information such as goals. Some providers such as allied health professionals were not able to gain access to this system, and primarily used demographics, risk behaviors and clinical monitoring such as HbA1C levels to facilitate their service delivery and communication with other providers.

### How information is used to support care

Information, both electronic and paper-based, was used in a number of ways. Participants described the electronic health record as “considered to be fully patient controlled,” with patients able to access the information held in the record and in control of whom else could access it. When a care plan and Team Care Arrangement was developed, patients consented to allied health professionals accessing their record. Patients were not able to enter data directly into the record, but information such as goals and tasks were developed by the patient in conjunction with their health care team and then entered.

One of the novel aspects of the electronic record, to which the greatest number of GPs had subscribed, was the patient access to their pathology results. For patients in a practice with the electronic record, doctors could enter their comments on routine laboratory tests in the record, allowing patients to view their results without the need for a phone call or unnecessary appointment.

Templates were commonly used for the entry of care plans and program progress information from allied health professionals, but participants reported that some in-practice systems did not communicate directly, and the need to enter data into each system separately was a burden.

… one of the issues that we have faced… is having to enter data in multiple spots…And that’s been an issue, because I just don’t have the time within one session to enter the information into the Care Plan and then enter the information into the software, and then there’s an Annual Cycle of Care…You know, there’s just multiple places for it to be entered… [C2I2]

Despite the availability of the care management information in the electronic record, much information to support multidisciplinary care was still reliant on manual transfer due to the communication requirements for referrals under the MBS. Referrals and reports were usually printed to be delivered between professionals by the patient or by fax. It was hoped that the referral requirements could be incorporated into the electronic record but this had not yet occurred. One practice had a successfully operating diabetes clinic that regularly followed up patients on care plans every six months, but the participant described the administrator, whose sole responsibility was to generate the recalls and make the appointments with patients, as the “lynchpin” of the system.

### Influence of governance, confidentiality, data ownership and standards

Data ownership was unclear. Although the electronic record was patient controlled and some participants saw the patient as the custodian, the consent processes attributed ownership to the administrators of the software. The consent had been developed with legal advice due to variations in state law regarding privacy. Other participants saw each practice, and the general practitioner in particular, as the custodian and owner of its own data.

The main influence of practice governance and privacy arrangements on information use was that front end staff did not have access to patient results in the practice management software. Part of the daily practice management instead involved clinical staff processing results and generating a list of patient recalls for follow-up.

### Influence of financial incentives and quality improvement programs

One participant felt that patient information was used more to trigger specific Commonwealth payments to the practice for chronic disease management work than for coordination and management of care. For payment to be triggered, the doctor needed to complete a chronic disease care plan. Additional payments may be triggered by additional work, such as completing an annual ‘cycle of care’ for people with diabetes. Chronic Disease Management payments were recognized as an important part of the profitability of the clinic. However in terms of patient care, information for the care planning process was separate from the information needed to generate recalls and manage care generally and was held in another part of the practice information system. Other participants expressed similar views, describing the information collected and use of the software tools in terms of what was needed to generate practice incentive payments but acknowledging that generation of a care plan did not guarantee action, particularly where patients were responsible for following up.

Well the patient can present to us, and we can do the Mental Health Plan – we can bill Medicare, and that’s paid. We’re paid. But then we’re paid for doing that, and whether the patient proceeds with that is another matter, isn’t it? [C2I4]

It was also suggested that the uptake and full use of the electronic record had been hampered by the lack of a specific incentive payment for doctors. The duplicative processes involved in maintaining the electronic record as well as clinical notes and cycles of care information meant that without payment for the extra time spent, fewer clinicians were interested in using the record.

There was some use of data aggregation for quality improvement to review and monitor practice performance on chronic illness management. However, the standardized entry of information required for aggregation was an ongoing problem, with one practice manager commenting that she was reminding the doctors on a weekly basis to be timely and consistent with data entry so that quality improvement statistics were accurate.

### Case Three

The primary problem the large scale SEHR aimed to address was the availability of information for a highly mobile and remote population. This government funded initiative had successfully automated the process of the entry of information from clinical records and as described below, gave patients control over what information was contained in the record and providing a rich resource for health professionals to access.

### Scope of information used

Participants described the information contained in the SEHR as a “scraping” of the information recorded during a consultation. With the patient’s consent, at the close of each consultation, a summary of clinical information such as test results, diagnoses, allergies, immunizations and medications could be added to the SEHR by checking a dialogue box in the software.

### How information is used to support care

Participants stressed that the purpose of the SEHR was to provide effective linking of primary health care to remote and highly mobile populations, removing the need for patients to tell their story repeatedly to different health providers and sign multiple consent forms to release records from previous provider; and by systematizing, maintain a current and accurate record of health status and medications that can be accessed immediately.

Well those who use it, and use it well, they’re not required to ring up, because the information’s there and available. And so we have some… well a very good user… if anybody rings him or his clinic for information, he just says, “Look up the Shared Electronic Health Record, it’s there. Help yourself.” [C3I2]

Clinicians had a unique identifier that allowed access to the records, either through enabled clinical systems or via an Internet portal and the use of the records was monitored by the government organization overseeing the initiative. The consent model provided patient control over the information added to the record at each consultation but the patient did not have direct access to the record.

The SEHR did not contain information such as care plans, although there were future plans for a shared electronic care plan.

### Influence of governance, confidentiality, data ownership and standards

As for the other organizations already described, the governance arrangements concerning ownership of the data were unclear. Participants described the consent model as patient control, but suggested that the data belonged to the clinician or organization entering it. Patient SEHRs were identified by a unique number and connected to clinical records at the practice. Clinicians also had a unique identifier for authentication in the system. Clinicians were only able to access the record if their digital authentication was confirmed by the system. The digital authentication system was able to recognize people trying to access a record who did not have permission, such as practice administration staff, and barred access.

So we have direct link between an icon embedded into these systems, so that if the clinician is registered with us, because when they try to get the access to the SEHR through that icon, we know what their user code is, we recognize that the organization has allowed them access and they will get direct access to the index page of a Shared Electronic Health Record. [C3I2]

The clinicians’ clinical records were described as “feeder systems” for the SEHR, with summary information added to the SEHR using a tick box prompt with the patient’s consent when the clinical record was completed at the end of a consultation. Patients could elect to have sensitive information omitted from the SEHR.

### Influence of financial incentives and quality improvement programs

As an information sharing system, the SEHR was not connected with payments, incentives or quality improvement programs. Information in the record was used at the patient level only, with no aggregation or extraction for other purposes other than to estimate community registration rates. Participants commented that the lack of incentive payments for involvement in shared records made it difficult to attract GPs to the system initially, but that the efficiency gains went some way to overcome this. Evidence for the influence of the SEHR on clinical outcomes had been positive but mostly informal.

Participants considered the SEHR a rich and comprehensive record and reported fighting to maintain its integrity with the introduction of the national PCEHR, but saw the two systems as working in parallel.

### Case Four

As a national private sector organization offering telephone-based triage, coaching and disease management, information collection and transfer was integral to the Case Four business model. With extensive guidelines, decision-support systems and illness management plans, information was used extensively within the organization’s systems, but rarely flowed outside the company.

### Scope of information used

Of the four organizations in the study, Case Four participants described the widest range of information collected routinely, largely due to their services being telephone-based and often utilizing decision support systems. Providers on the triage systems collected information on demographics, clinical history, usual health providers if possible, symptoms, and conducted a risk assessment, particularly for mental health triage.

For the programs focused on health coaching for chronic disease and mental health relapse prevention, the information used also extended to risk behaviors, health assessments, self-management activities, health literacy and motivational interviewing to assess readiness for change.

### How information is used to support care

Across the various branches and programs of the organization, information was collected, transferred and used in standardized ways to support the care provided. Most of the telephone triage services used decision support systems and protocols to ensure the care a caller received was consistent and delivered according to practice guidelines. The nurse triage line was supported by 500+ guidelines and nurses received extensive on-the-job training and regular auditing and support to ensure that the service conformed to its guidelines. Occasionally triage line staff referred to and shared case information with other services such as the GP after-hours telephone service and a very small number of community health practices, but this was not common. The exception was when there was a high risk, such as serious suicidal ideation expressed by a caller.

For the chronic disease management and mental health relapse prevention programs, participants described the use of templates and protocols to develop individualized health action plans (chronic disease) and relapse prevention plans (mental health). Clients were referred into these programs by their private health insurer, who provided only basic demographic and contact data to the Case Four organization and received only aggregated program level data in return. Clients were screened for program suitability by a call centre operator and enrolled in a program by a health professional if appropriate. Information on health assessments, goals, self-management and health literacy was collected and a plan developed with the client. Programs typically continued over a number of months, but they were not intended to be a substitute for other medical and health care and their scope was tightly defined. With the consent of the client, summaries of progress and signs of relapse were shared with the regular treating professional and on complex mental health cases, input was also sought from the treating professional.

If we need to notify the treating professional that the person is becoming unwell we obviously do that by phone as well. We do, there is some sending of… as I said of letters, so we send a letter to the professional to let them know we’re involved…So look there’s not a lot of contact but there is, often at the beginning, particularly if it’s a more complex case we’re eager to get the opinion of the treating psychiatrist, if they’re, with some patients are more high risk and so that’s when we get more involved but it would only be, often one or two calls during the course of the year. [C4I1]

Participants were careful to point out that the information used to support the care of clients and to provide feedback to the professionals delivering the services was separate to that provided to the contractors and private health insurers. Information provided to these latter was aggregated data on enrolments, overall risk reduction for programs and client satisfaction as described below.

### Influence of governance, confidentiality, data ownership and standards

As a large company that had amalgamated with a number of other providers and services, participants reported that the governance arrangements for the management of information were complex and indistinct. All information was electronic, primarily stored in a data warehouse. It was suggested variously that data ownership could be attributed to the clients, the case managers, the clinical quality managers, the data warehouse and analysts and the health bureaucracies purchasing the programs under contract.

The flow of information between professionals within the organization and to others outside the organization, such as clients’ regular treating professionals, was governed by client consent in accordance with state-based and national privacy laws. When contracted to deliver a program or service, standard agreements that satisfied government reporting requirements and privacy legislation were signed.

We are… work under the requirements of the privacy legislation and the national privacy principles, and so that really in regards to who owns the information, and how information is disseminated, collected, stored etcetera, is fairly standard. [C4I5]

Participants noted that despite the computerization of all records, the use of secure messaging and web conferencing to support service delivery and quality improvement and the storage of data in a data warehouse, reporting was not automated and was cumbersome to carry out and letters were still used to communicate with treating professionals and the client.

### Influence of financial incentives and quality improvement programs

As an independent contractor providing self-contained programs and triage services, financial incentives were not a strong influence on the use of information in Case Four. However, one participant did note that programs into which private health insurers referred, such as the chronic disease management program, were aimed at reducing the costs to private health insurers of poorly managed illness. Clients were referred to these programs based on their claims history and although individual progress was kept confidential, reporting to the insurer on overall performance included aggregated data on reduction of risk factors and improvement in self-management.

Information use for quality improvement processes was a very strong theme for Case Four. There was particular emphasis on the use of auditing and ongoing quality training to ensure that the services delivered were consistent and of high standard, driven by the contract environment.

Calls were audited regularly and monitored to ensure that customer service quality standards were met, that clinical guidelines were followed and for the management programs, that the health professional tried to engage the client in behavior change. As the programs were designed around evidence-based interventions, participants said there was an assumption that they were effective and that clinical outcomes were therefore less often measured and reported to contractors.

So therefore the two elements are evidence that the way that care is delivered is quality, it ensures clinical quality of service delivery, and evidence that the service is delivered faithfully and efficiently. The evidence of the outcomes for that particular service takes care of itself. [C4I5]

### Other themes

Participants for Case Four saw two roles for their organization in coordination of care. The telephone triage services were focused on providing access and referral to appropriate services, easing pressure on emergency departments and facilitating clients’ access to care. Particularly in rural areas, participants believed that the mental health triage line acted as a crucial part of the community mental health services.

For the health coaching and disease management programs, participants suggested that the Case Four providers acted in a case management type role, helping to improve health literacy and self-management and reduce modifiable risk factors in conjunction with regular care from treating professionals.

## Discussion

### Does information continuity exist and does it support coordination?

The collection and use of information appeared to be determined by the job in hand regardless of the business model used by the organization. However, the two sets of organizations operating from a private enterprise model were more specific about what information they collected and how, and were more aware of the costs of any change.

Three forms of information continuity emerged from these interviews:

1. Active communication and the “carrying” of information from place to place, as in Case One.

2. Information progressing one stop from the source, or continuity within a work-defined environment. With rare exceptions such as risk of suicide, information was retained within the area for which a business held it, such as in general practice or a specific program in Case Four.

3. Information accessible to multiple professionals without individual professionals actively seeking to enhance coordination of care, shown by Cases Two and Three.

Thus, according to Haggerty and colleagues’ definitions [9], the Case One model relied heavily on management continuity to facilitate both relational and information continuity with the role of the liaison nurses as brokers and bridges crucial to the coordination process. Likewise for Cases Two and Three, despite the existence of a powerful electronic record, information flow was still patchy and heavily reliant on people’s management practices to coordinate care. This observation is consistent with the findings of both Crooks and Agarwal and Reid et al., who contend that the three forms of continuity are interdependent and complex and dependent on providers, patients and the technology all playing their respective roles [10,11].

Case Four seemed to operate with a sophisticated electronic system of information recording and use but was subject to blockages in the effective exchange of this information with providers outside their own systems. This was partly governed by the application of the privacy laws and strict policies regarding confidentiality and consent. It was also driven by the contractual nature of the programs being delivered, making much of the information aggregation and sharing more about business processes than coordination of care. Similar barriers were common in Cases One and Two, where concerns about the propriety and indeed, legality, of data sharing accompanied an absence of any clear responsibility for data management and custodianship.

Few of the people interviewed indicated that information collected led to any form of collaborative team work, or to any active coordination of the care of a person with chronic illness. One participant expressed considerable frustration at not being able to get information from their local practice and further, not being able to get anyone in the practice interested in the information that they thought it was important to share. This is a clear example of the failure of the provider experience of continuity, as this allied health professional was unable to access information he needed and did not feel his care was being recognized [10]. Most participants expressed no interest in or awareness of activities outside their own sphere or set expectation, although providers in Case Four recognized that a client’s usual treating professional may need to be kept informed. This is consistent with the findings from other studies described in Waibel and colleagues’ meta-synthesis, where information continuity was highly valued but often hampered by failures in cross-boundary communications, resulting in patient frustration [13].

### What is required to support coordination?

The overall picture that emerged across all four cases was that whilst accessibility and continuity of information underpin effective care, they are not sufficient for coordination of care for complex conditions. Similar to Reid et al’s observation that the simple transfer of information is not sufficient [10], our study found that the active involvement of a care provider in a case management-type role was required for information continuity to be associated with collaborative care. Shared information reduced unnecessary repetition and provided health professionals with the opportunity to access records of care from other providers. However, as previously observed by Bodenheimer [14], coordination tended to be described in terms of the active involvement of a person in care rather than the passive availability of information.

This has implications for e-health initiatives in health reform, which in Australian policy documents include the expectation that enabling access to information will lead to better coordination of care [19]. While this expectation is justified in part by this research, an expectation that access to information is universal, or will be used, is not. It was reported in our study that substantial numbers of general practitioners are not IT connected, and have little intention of becoming so, despite substantial support to do so. That these practitioners were in an area of disadvantage raises the question of whether other disadvantaged areas, and the people in them, will be similarly left behind, compounding the existing inequalities in health. Similarly, it is clear from this research that not all providers will be part of the system: access to information relies in some measure on patient permissions, but in the case of many primary care providers, on being seen by the principal practitioners as part of the contributing team. The confusion over data ownership and confidentiality is a further complication. Thus, whilst initiatives such as the PCEHR may increase the availability of information to providers across the health system, potentially increasing efficiency and safety, and improving the quality of decision making, our findings suggest that this will only be the first step in achieving the aim of improving coordination of care for chronic conditions in Australia [19].

This study confirms other findings that success (and failure) in health information systems is shaped by the degree to which they are responsive to the needs of patients, carers and health care providers. Greenhalgh and colleagues have argued that systems that are not aligned with these needs, but designed top down according to static standard protocols, are unlikely to be taken up by patients and clinicians [20]. Instead, progress towards information continuity must start from a dynamic understanding of systems, as ‘components of a socio-technical network’. In each of these case studies the forms of information exchange followed patterns shaped by local factors. With the two less structured systems, their limitations – and relative achievements – were shaped by institutional factors: the degree of trust between organizations requiring a mediating agent (Case One), and the business models of participants (Case Two). The two cases with more developed informational flows started from quite different problems: widely dispersed practitioners in a remote region, where better communication relieved much of the burden of health care (Case Three) and a business model that could use access to large data bases and started from a close alignment of incentives to drive down costs by improving prevention and patient care (Case Four).

### Limitations

Due to limited time and funding, this study was conducted with a small number of participants in a small number of primary care models. Participants were identified by managers within the organizations based on a description of the study’s focus. These factors may have limited the findings both within and across organizations by only reaching a specific type of participants.

## Conclusion

The availability of information is not sufficient to ensure continuity for the patient or coordination from the systems perspective. This study has identified a number of challenges facing health services for coordination of care and for the development of effective policy to support it. Consideration must be given to the impact of technological limitations ranging from a complete lack of practice computerization to incompatible software systems, coupled with complex data ownership and confidentiality issues. Policy directed at information continuity must give consideration to the broader ‘fit’ with management and relational continuity for coordination of care to be achieved.

## Abbreviations

GP: General Practitioner; HbA1C: Hemoglobin A1C; MBS: Medicare Benefits Schedule; PCEHR: Personally Controlled Electronic Health Record; SEHR: Shared Electronic Health Record.

## Competing interests

The authors declare that they have no competing interests.

## Authors’ contributions

MB participated in the design of the study, conducted interviews and analysis and drafted the manuscript. KG participated in the design of the study, conducted interviews and analysis and contributed to the drafting of the manuscript. IM and JG conceived the study, participated in design and analysis discussions and contributed to drafting of the manuscript. RW conceived the study and participated in design and analysis discussions. LY conceived the study, participated in design and analysis, conducted interviews and contributed to drafting of the manuscript. All authors read and approved the final manuscript.

## Pre-publication history

The pre-publication history for this paper can be accessed here:

http://www.biomedcentral.com/1471-2296/14/34/prepub

## Supplementary Material

Additional file 1 Improving coordination through information continuity interview protocol. Click here for file
