# Supplementary material for: Unlocking information for coordination of care in Australia: a qualitative study of information continuity in four primary health care models
Source: BMC Fam Pract. 2013 Mar 13;14:34. doi: 10.1186/1471-2296-14-34 (PMC3605301; doi:10.1186/1471-2296-14-34)
Supplement: Additional file 1 — Improving coordination through information continuity interview protocol. [file 1471-2296-14-34-S1.pdf]

Improving coordination through information continuity interview protocol

Key research interests

1. What is the scope and type of information stored, transferred and used between providers to support coordination of clinical care?

2. How is information used by managers, clinicians and clients in different models to support disease management, multidisciplinary care, care planning and case management?

3. How do the governance arrangements, client confidentiality, data ownership and standards support or inhibit the use of information?

4. How are financial incentives and quality improvement programs used to stimulate the use of information in coordination?

Question guide

PART A For clinicians

1. **<u>Clinical care</u>**

What type of information do you collect to support the following activities?

- Patient assessment
- Evaluate health outcomes

Thinking about a care plan/ chronic disease management program, what information do you need to:

- identify the appropriate providers for the care plan
- communicate with participants to ensure information transfer
- Execute the care plan - ie. deliver the coordination intervention
- Monitor the care plan

This may be information from different providers, demographic and social information about the patient from a home or environmental assessment, medical information

Prompt

Types of information might include:

- Non-clinical – patient demographics, living situation, risk behaviours, (smoking, nutrition, activity, exercise)
- Clinical information
- Satisfaction/patient preferences (which services used and preferred– GP, allied health, pharmacy, specialist)

Data records, storage and transfer

How is clinical data recorded, stored and transferred?

- Paper records or electronic?
- Where is the information stored?
- How is information transferred between providers?

How do you maintain confidentiality with care plans and teamcare?

Data custodian

Who is the custodian of the data/shared record?

Who has responsibility for linking care events and communicating with providers?

When constructing care plans, who is the case manager and how do they work with the care plan?

Templates and data entry

Do you have templates for data collection (such as a care plan?)

- Has that been developed by the practice management software?

Do you have problems with software management? (eg. Restrictions on linking item numbers, collecting information required for billing etc.)

Is data entered under specific codes? If so, what are the codes?

How is information standardised to ensure accurate and comparable entry?

Is training provided for data entry?

Access to information in the care plan

Which providers have access to the records/ care plan?

- Partial or complete? – which components?

Does the patient have and control access to information in the records/care plan?

Does the patient approve the record/care plan?

Are there barriers to using that information for the purposes outlined?

How long are consultations under this arrangement?

Billing and costs

Does the template generate the information required for medicare items?

Who is responsible for claiming care planning or other items from medicare?

How is that income distributed?

Is the care plan costed?

Does the care plan contain a comprehensive record of services received?

- If so, how do you ensure this?

PART B For managers

Is patient information (collected at the clinical level) used to support any of the following activities?

- quality improvement (delivery of best practice care),
- business management (income generation and billing etc),
- planning,
- community engagement
- costing

- If so, how is the information used?

How are incentives, such as MBS item payments, used in the [practice/division/area] to stimulate improvements in coordination?

Are there incentives other than financial incentives that clinicians respond to?

- If so, how is the information you collect used to support these?

Is information used to assess improvements in

- efficiencies
- control costs
- improve disease outcomes
- quality of care
- patient satisfaction?

Prompts

- o MBS billing for care planning,
- o health checks,
- o team care (get the right MBS items),
- o prescribing
- o or for reporting on a program of some sort (e.g., Collaboratives)
